# The Time Course of Perceptual Closure of Incomplete Visual Objects: An Event-Related Potential Study

**DOI:** 10.1155/2020/8825197

**Published:** 2020-10-06

**Authors:** Chenyang Liu, Sha Sha, Xiujun Zhang, Zhiming Bian, Lin Lu, Bin Hao, Lina Li, Hongge Luo, Xiaotian Wang, Changming Wang, Chao Chen

**Affiliations:** ^1^North China University of Science and Technology, Tangshan, Hebei 063000, China; ^2^Depression Treatment Center, Beijing Anding Hospital, Capital Medical University, Beijing 100088, China; ^3^Zhonghuan Information College Tianjin University of Technology, Tianjin 300380, China; ^4^School of Artificial Intelligence, Xidian University, Xi'an 710071, China; ^5^Key Laboratory of Complex System Control Theory and Application, Tianjin University of Technology, Tianjin 300384, China; ^6^Academy of Medical Engineering and Translational Medicine, Tianjin University, Tianjin 300072, China

## Abstract

Perceptual organization is an important part of visual and auditory information processing. In the case of visual occlusion, whether the loss of information in images could be recovered and thus perceptually closed affects object recognition. In particular, many elderly subjects have defects in object recognition ability, which may be closely related to the abnormalities of perceptual functions. This phenomenon even can be observed in the early stage of dementia. Therefore, studying the neural mechanism of perceptual closure and its relationship with sensory and cognitive processing is important for understanding how the human brain recognizes objects, inspiring the development of neuromorphic intelligent algorithms of object recognition. In this study, a new experiment was designed to explore the realistic process of perceptual closure under occlusion and intact conditions of faces and building. The analysis of the differences in ERP components P1, N1, and Ncl indicated that the subjective awareness of perceptual closure mainly occurs in Ncl, but incomplete information has been processed and showed different manners compared to complete stimuli in N170 for facial materials. Although occluded, faces, but not buildings, still maintain the specificity of perceptual processing. The Ncl by faces and buildings did not show significant differences in both amplitude and latency, suggesting a “completing” process regardless of categorical features.

## 1. Introduction

In everyday life, visual objects are usually shaded and even occluded, making visual content inputs to human visual systems incomplete. We have to be accustomed to complementing views blocked by leaves, perceiving visual scenes in dust and recognizing a friend with a face mask.

Loss of information in early visual processing stage has to be completed following some rules in order to form a stable and holistic perception prior to cognitive stage, which facilitates recognizing objects quickly and accurately. This completion stage between sensory and cognitive process is described as perceptual organization according to the theory of Gestalt psychology [1]. Specifically, the process to deal with incomplete, incoherent, and intermittent visual content is considered as perceptual closure against lack of physical closure [2], which may start several subprocesses to make perception complete, such as element integration, contour detection and formation, feature binding, and template matching. Deficits in perceptual closure results in failure in object recognition, misidentification of emotion, and even hallucination, which is popular in elder people and patients with neuropsychiatric disorders [3]. Actually, perceptual closure is not only a processing stage but also reflects the completion result of incomplete information. It not only plays very important roles in processing visual information in human brains, but also crucial in inspiring developing intelligent algorithms and artificial neural networks. However, the time course and the neural mechanisms of how occluded objects are processed in our brains are still not fully understood.

When we see incomplete visual images, brain areas in the occipital, temporal, and parietal lobes participate in the completing processes. In functional magnetic resonance imaging (fMRI) studies, lateral occipital complex (LOC) and posterior intraparietal region was found to be more activated by incomplete stimuli than complete stimuli in the perceptual closure task [4, 5]. Early visual cortex and fusiform areas in inferior temporal cortex are activated rapidly, and then incomplete information in visual content is predicted which is conducive to subsequent target recognition [6, 7]. The bottom-up processing starting from low-level visual features is also regulated by top-down processing, as subjective perception is usually affected instructions and visual experiences because of the involvement of frontal networks [8]. In addition, intraparietal sulcus (IPS) and inferior parietal cortex (IPC) in dorsal visual stream also interact with temporal and occipital cortexes in ventral steam when dealing with stimulus degradation by noise [9]. However, the time course of perceptual closure is still worth for further investigation.

EEG has higher time resolution than fMRI, so this technology can provide fine description of the time course of sensory and perceptual processes of incomplete visual information. The EEG signal evoked by visual or auditory stimuli is categorized to event-related potential (ERP). In ERP studies, P1 and N1 are two typical components related to visual sensory and perceptual processing [10]. P1 starts at about 60–90 ms after visual stimulation and peaks at 100–130 ms, which can be observed at electrodes around bilateral occipital regions. It is believed to reflect the early sensory process of low-level visual information [10]. N1 is a component that begins at 130 ms after the presentation of facial and other visual objects and peaks at 160–180 ms [11, 12]. N1 can be evoked by many visual stimuli such as cars and houses and that by human faces is specifically named N170 [11]. N1/N170 is sensitive to object categories, and the amplitude of N170 by faces is much larger than N1 by nonface objects [10]. Given that faces usually contains more elements and involves integrating more complex features than other objects, the differences in N1 and N170 may be ascribed to the organization manners of visual features, and N170 corresponds to the processes of structural encoding of global visual features of faces [13, 14]. This implies that the neural activities producing N1/N170 is more likely to be responsible for perceptual organization of various features rather than the initial sensation of low-level information.

In the case incomplete visual objects, a subsequent component named negativity of closure (Ncl) could be evoked except for the two earlier components above. Previous studies indicated that when people are viewing incomplete line drawings, a negative wave that starts at 220–230 ms and reaches a peak at 290–300 ms can be found mainly in the occipital or occipito-temporal lobe [15–17] and can be localized to the electrophysiological activities in LOC [18]. This Ncl component is believed to reflect the process of visual completion instead of visual representation, but it needs extensive exploration on different styles of stimulus before wide acceptance [19, 20].

To sum up, the time course of neural activities for incomplete objects is still unclear. Although the characteristics of P1 and N1/N170 have been repeated by many studies, they are mostly evoked by complete visual objects and the conclusions cannot be automatically generalized to incomplete objects. There is still lack of evidence about whether N1/N170 or Ncl is more important for perceptual closure. And even whether incomplete information has been complemented or at least detected in the temporal stage around P1 is worth experimental studies. In addition, incomplete objects express with several styles, including occluded objects, virtual contour consisting of discrete elements, and noisy images with different visibility. Occluded faces and familiar nonliving objects have seldom been studied since they are better examples of perceptual closure than contours.

To answer the above questions, a new experiment paradigm and new visual stimuli consisting of a series of occluded faces and buildings were designed to explore the differences in three different ERP components. The stimuli contains two exemplars of visual objects, and by comparing the occluded edition of them with the intact edition, ERP under occlusion conditions and intact conditions could be evoked and thus enable systematic comparison. The sequential change of differences in ERP amplitude and latency also helps reveal the time course of perceptual closure. All the efforts above could provide solid support when we are trying to reconstruct object recognition ability for elder people and to construct neural networks with human-like intelligence following the neural mechanisms of human visual systems.

## 2. Methods

### 2.1. Participants

A total of 22 healthy subjects were included in this study (Table 1). All subjects had normal vision or corrected visual acuity. They fully understood the experimental task and signed the informed consent. This study was approved by the Ethics Committee of Beijing Anding Hospital affiliated to Capital Medical University.

### 2.2. Stimuli

**Figure 1:**
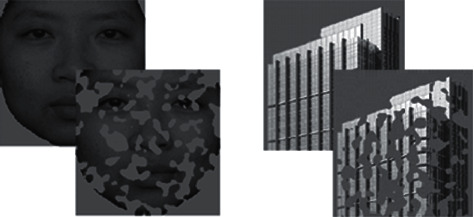
ERP waveforms of occluded and intact faces.

The facial materials used in this study were quoted from the Chinese Standard Emotional Face Picture Library. Natural neutral facial pictures were selected as stimulating materials in this experiment. 40 Male and 40 female faces were used. 40 building pictures were collected from the Internet and edited by the authors of this study. Adobe Photoshop was used to randomly occlude the whole experimental material, and the average occlusion ratio was 41.5% of the whole picture (Figure 1).

### 2.3. Experimental Paradigm

**Figure 2:**
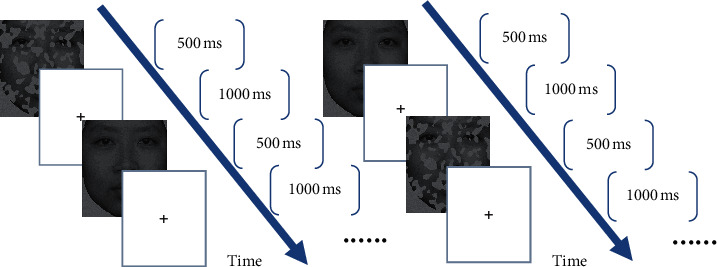
The experiment procedure of the complete-occluded object comparison task.

In this experiment, the experimental stimulus was presented by E-Prime 2.0, faces and buildings were presented on a LCD monitor in two separate sessions, and each session consists of 140 trials. In each trial, the subjects were presented with two pictures (one complete picture and one occluded picture) in random order. Each stimulus picture lasts 500 ms in screen and was followed by a blank screen with a “+” in the center. The task of the experiment for the subjects was to judge whether the two pictures (occluded and complete) presented can be recognized as the same picture and to feedback the judgment by pressing “F” or “J” on the keyboard. When the second picture is presented, subjects press the keyboard to feedback. The ISI (interstimulus interval) was 1000 ms, and ITI was 2000 ms (Figure 2). Before the experiment was officially started, there were 10 practice trials to ensure that the subjects were clear about the task of the experiment.

During the experiment, EEG data were recorded by EGI 128-channel EEG system (Electrical Geodesic Instrument, USA). The sampling rate was 1000 Hz, and Cz was selected as the reference channel. During the experiment, the scalp resistance of each subject was guaranteed to drop below 50 kΩ.

### 2.4. Data Analysis

The EEG data were analyzed using EEGLAB 13.5.4b (http://sccn.ucsd.edu/eeglab/), an open-source signal processing toolbox on Matlab platform and programs developed by the authors to preprocess the EEG data including remove eye movement noise. The sampling rate of the EEG data was downsampled to 250 Hz, filtered by 0.1–40 Hz by a bandpass filter, and the average of EEG from all channels were used as reference. Principal component analysis (PCA) and independent component analysis (ICA) were used to decompose EEG signals into 20 components in usual, and those related to eyeblinks and eye movement noise were identified visually by the author and removed manually. Then EEG data of all trials were epoched into segments of −200 to 500 ms relative to the stimulus presentation onset. Segments corresponding to four stimuli types (complete\occluded faces and buildings) were averaged, and ERP waveform was finally extracted. The amplitude and latency of peaks in three ERP components P1, N170, and Ncl were selected for statistical analysis in SPSS 19.0.

## 3. Results

**Figure 3:**
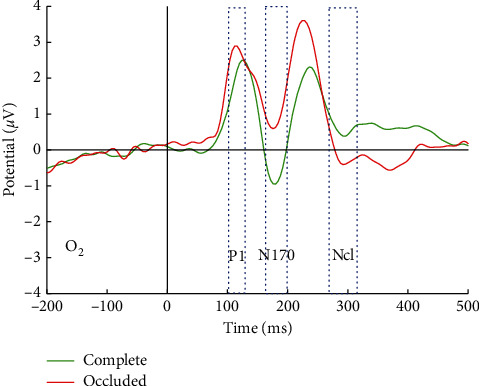
ERP waveforms of complete and occluded faces.

**Figure 4:**
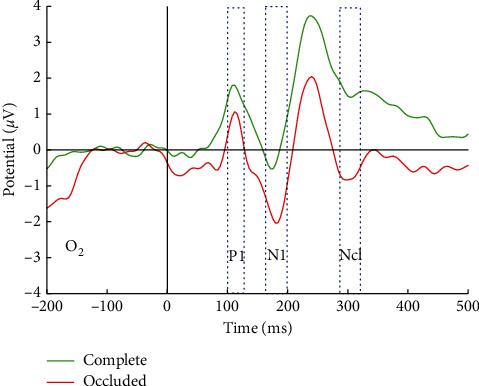
ERP waveform of occluded building and complete building.

In this study, P1 and N170 could be elicited by both complete and occluded faces in commonly accepted visual areas, i.e., right occipital lobe (corresponding to O_2_ in 10–20 EEG electrode systems) (Figure 3). For buildings, P1 and a smaller component N1 (which is thus not named as N170 for nonface objects) could also be observed in Figure 4. The differences of amplitude and latency in ERP components between complete and occluded visual stimuli were further analyzed.

In the case of face condition, the amplitude and latency of P1 component were not significantly different (Table 2) between complete and occluded conditions according to the results of paired-sample *T*-test (*p* > 0.05). In addition to faces, significant differences could be found in neither latency nor amplitude in P1 component between occluded and complete buildings. One possible explanation is that P1 component corresponds to the early stage of initial processing of low-level visual information, and object category and even object completeness detection have not started at P1 stage.

Then the amplitude and latency of N170 component between complete and occluded faces were both significantly different (*p* < 0.05, Table 2). The peak latency of N170 for occluded faces was 4 ms early than that for complete faces, and the amplitude of N170 for occluded faces was significantly more positive than that for complete faces, suggesting occluded objects may be processed more quickly because of smaller amount/loss of visual information and resulting in a less sufficient structural encoding process (1.57 *μ*V smaller in N170 peak amplitude). However, the effect in N170 for occluded stimuli could not be observed in N1 by occluded buildings. According to the results in Table 3, the peak amplitude values of N1 by occluded buildings were not significantly more positive than those by complete buildings (*p* > 0.05). N1 by occluded buildings occurred later in peak latency, but the differences were not statistically significant.

The ERP component Ncl could be evoked by both occluded faces and occluded buildings, not by complete visual stimuli. The findings in this study support that Ncl is specific to incomplete visual object and thus named negativity of “closure” in early studies. Unlike the abstract object made by line drawings used in previous studies (the unoccluded object by line drawings still look “incomplete”), real face and buildings were selected and occluded in this study. Hence, further evidences were provided supporting a common “complete” progress for all visual objects including faces and nonface objects happening around 200–300 ms after visual presentation.

## 4. Discussion

How incomplete objects are retrieved, complemented, and processed remain a challenge in both cognitive neuroscience and artificial intelligence. Intact and occluded visual objects may activate different neural mechanisms in initial representation stage, perceptual organization stage, or relatively late stage.

This study designed a new paradigm and new stimuli to investigate the process of perceptual closure by comparing the ERP differences between occluded and complete conditions. The visual materials were from the same collection of face and building images, and thus they had the same size and the same content, except for the degree of occlusion. All of them were presented to subjects in random order, and all the evoked EEG responses were used to generate ERP by grand average. Hence, differences in ERP could be ascribed by different experimental conditions, i.e., occlusion or completeness. Another advantage of ERP lies in the high temporal resolution which could separate different stages of object completion and thus help exploit which stage contribute more to the visual completion of multiple categories of objects. All the investigation above empirical information guide scientists and engineers to design more intelligent and efficient hardware or algorithms for visual information processing.

When does perceptual closure occur, at the very early stage or at a relatively late stage of visual information? This question has not been fully answered, leaving much confusion when experts majored in computational intelligence are trying to design an artificial network to identify or recognize visual objects inspired by the human brains. According to previous studies, occluded objects are believed to have been processed in the stage corresponding to Ncl component [15, 18]. In this study, similar conclusions could be drawn since an ERP component Ncl specific to perceptual closure could only be evoked by incomplete stimuli; moreover, the stimuli categories were expanded from abstract line drawings to real faces and buildings.

When tracing back to an earlier stage corresponding to N1/N170 component, that is, around 80 ms earlier than Ncl, we found that incomplete visual information had been processed at least for faces. Incompleteness may be detected first because of earlier latency in N170 component for faces and then starts a subsequent complementation process around 170 ms which is believed to correspond to the structural coding process in Bruce–Young model and newly revised model [14, 19, 21]. Insignificant differences in either peak latency or amplitude in P1 component indicated that occluded and complete images were not distinguished at this stage because it is a too early stage that may only participate in initial low-level representation of visual information to encode the global wholeness [22].

In addition, in the processing stage corresponding to N1/N170, occluded objects of different categories may be processed and complemented in different manners. For face images, the perceptual closure may occur earlier than buildings as larger and significant differences could be observed in the N170 by faces, not in the N1 by buildings. The results in this study also showed that the process of perceptual closure is highly dependent on the underlying “template” or visual experiences [23, 24]. We are all experts in recognizing faces but not in buildings, so specific neural networks or modules to faces in visual cortex may have been evolved billions of years ago. As it has been shown in many literature studies, the N170 by faces is so distinct that its peak amplitude is usually much larger than the N1 and thus named by a new component [10, 11]. Even for occluded face pictures, the specificity in faces is still maintained.

Then a common process may occur subsequently regardless of the category of visual objects. Following N1/N170, Ncl component could be evoked by both incomplete faces and incomplete buildings, the waveform by both stimuli appeared similar, and both the amplitude and latency values of Ncl were not significantly different between faces and buildings. All the findings above indicate that there is a common “complete” process for all visual objects. Prior to this stage, visual objects from different categories are represented by neural activities distinctly, and low-level features are extracted, integrated, and organized according to the visual template of each category. It is thus prone to find category-specific perceptual closure ERP features in N1/N170 when processing occluded objects. When higher-level visual information resulting from perceptual organization is transmitted hierarchically to superior stage corresponding to Ncl compared to P1 and N1, it normally involves a pure processing stage irrelevant of visual content input here. Thus Ncl may reflect a subjective awareness of incompletion and recover it consciously.

In future studies, more categories of visual objects will be used to support and to expand the neural mechanisms of perceptual closure found in this study. In related works, P300 ERP component was used for brain computer interface study [25–27], the ERP components in this study also can be used to build an affective brain computer interface. BCI also can be very useful for the elder people [28–31]. To improve BCI performance for daily-life use, advanced classification methods are required [32, 33].

Furthermore, the ability of perceptual organization in elder people and in patients with neuropsychiatric disorders, such as schizophrenia, ADHD, and ASD, could be systematically investigated to help exploit how the neural network in their brain should still work in spite of impairment in neural oscillation and neural plasticity and thus help build a more robust artificial neural network to all kinds of noise.

## Figures and Tables

**Table 1:** Information of subjects ($\bar{x}$ ± *s*).

	*N* = 24
Age	26.54 ± 5.469
Education years	14.33 ± 3.088
Gender (male/female)	12/12

**Table 2:** Comparison of the amplitudes and latencies of each component of incomplete and complete faces ($\bar{x}$ ± *s*).

		Occluded	Complete	*t*	*p*
Amplitude (*μ*V)	P1	2.914 ± 2.285	2.529 ± 2.259	1.009	0.326
	N170	0.551 ± 2.986	−1.019 ± 3.092	2.225	0.039
	Ncl	−0.562 ± 2.694	0.399 ± 2.554	−2.567	0.017

Latency (ms)	P1	116 ± 7.438	117 ± 9.418	−0.944	0.358
	N170	176 ± 6.591	180 ± 7.342	−2.418	0.026
	Ncl	299 ± 10.557	298 ± 12.681	0.266	0.793

**Table 3:** Comparisons of the amplitudes and latencies of each component of incomplete and complete buildings ($\bar{x}$ ± *s*).

		Occluded	Complete	*t*	*p*
Amplitude (*μ*V)	P1	1.004 ± 1.365	1.836 ± 1.243	−0.543	0.242
	N1	−2.135 ± 1.867	−0.683 ± 2.687	−0.856	0.365
	Ncl	−0.886 ± 2.330	1.445 ± 2.426	−3.293	0.003

Latency (ms)	P1	108 ± 8.355	106 ± 5.454	0.134	0.891
	N1	179 ± 5.856	175 ± 7.693	0.644	0.453
	Ncl	301 ± 7.503	304 ± 12.067	−0.816	0.423

## Data Availability

The experiment data are not available online for further research but available on reasonable request according to the policy of North China University of Science and Technology, Capital Medical University, and Tianjin University of Technology.

## References

[1] Wagemans J., Elder J. H., Kubovy M. (2012). A century of Gestalt psychology in visual perception: I. Perceptual grouping and figure-ground organization. *Psychological Bulletin*.

[2] Snodgrass J. G., Feenan K. (1990). Priming effects in picture fragment completion: support for the perceptual closure hypothesis. *Journal of Experimental Psychology: General*.

[3] Kurylo D. D., Pasternak R., Silipo G. (2007). Perceptual organization by proximity and similarity in schizophrenia. *Schizophrenia Research*.

[4] Freud E., Robinson A. K., Behrmann M. (2018). More than action: the dorsal pathway contributes to the perception of 3-D structure. *Journal of Cognitive Neuroscience*.

[5] Hegdé J., Fang F., Murray S. O., Kersten D. (2008). Preferential responses to occluded objects in the human visual cortex. *Journal of Vision*.

[6] Laycock R., Crewther D. P., Crewther D. P. (2007). A role for the “magnocellular advantage” in visual impairments in neurodevelopmental and psychiatric disorders. *Neuroscience & Biobehavioral Reviews*.

[7] Doniger G. M., Foxe J. J., Schroeder C. E., Murray M. M., Higgins B. A., Javitt D. C. (2001). Visual perceptual learning in human object recognition areas: a repetition priming study using high-density electrical mapping. *NeuroImage*.

[8] Yantis S. (1998). Objects, attention, and perceptual experience. *Visual Attention*.

[9] Darcy N., Sterzer P., Hesselmann G. (2018). Category-selective processing in the two visual pathways as a function of stimulus degradation by noise. *Neuroimage*.

[10] Kappenman E. S., Luck S. J. (2011). *The Oxford Handbook of Event-Related Potential Components*.

[11] Itier R. J., Taylor M. J. (2004). N170 or N1? Spatiotemporal differences between object and face processing using ERPs. *Cerebral Cortex*.

[12] Wang C., Zhang J., Li Z., Yao Li, Hu X. (2012). Combining features from ERP components in single-trial EEG for discriminating four-category visual objects. *Journal of Neural Engineering*.

[13] Eimer M. (2000). The face-specific N170 component reflects late stages in the structural encoding of faces. *Neuroreport*.

[14] Liu J., Harris A., Kanwisher N. (2002). Stages of processing in face perception: an MEG study. *Nature Neuroscience*.

[15] Grützner C., Uhlhaas P. J., Genc E., Kohler A., Singer W., Wibral M. (2010). Neuroelectromagnetic correlates of perceptual closure processes. *Journal of Neuroscience*.

[16] Stuss D. T., Picton T. W., Cerri A. M., Leech E. E., Stethem L. L. (1992). Perceptual closure and object identification: electrophysiological responses to incomplete pictures. *Brain and Cognition*.

[17] Doniger G. M., Foxe J. J., Murray M. M. (2000). Activation timecourse of ventral visual stream object-recognition areas: high density electrical mapping of perceptual closure processes. *Journal of Cognitive Neuroscience*.

[18] Sehatpour P., Molholm S., Javitt D. C., Foxe J. J. (2005). Spatiotemporal dynamics of human object recognition processing: an integrated high-density electrical mapping and functional imaging study of “closure” processes. *Neuroimage*.

[19] Zhao P., Li S., Zhao J., Gaspar C. M., Weng X. (2015). Training by visual identification and writing leads to different visual word expertise N170 effects in preliterate Chinese children. *Developmental Cognitive Neuroscience*.

[20] Jemel B., Pisani M., Calabria M., Crommelinck M., Bruyer R. (2003). Is the N170 for faces cognitively penetrable? Evidence from repetition priming of Mooney faces of familiar and unfamiliar persons. *Cognitive Brain Research*.

[21] Campbell R. (2011). Speechreading and the Bruce-Young model of face recognition: early findings and recent developments. *British Journal of Psychology*.

[22] Ganis G., Smith D., Schendan H. E. (2012). The N170, not the P1, indexes the earliest time for categorical perception of faces, regardless of interstimulus variance. *Neuroimage*.

[23] Koenderink J. J. (2015). Gestalts as ecological templates. *Handbook of Perceptual Organization*.

[24] Kimchi R., Hadad B.-S. (2002). Influence of past experience on perceptual grouping. *Psychological Science*.

[25] Jin J., Chen Z., Xu R., Miao Y., Wang X. Y., Jung T. P. (2020). Developing a novel tactile P300 brain-computer interface with a cheeks-stim paradigm. *IEEE Transactions on Biomedical Engineering*.

[26] Jin J., Li S., Daly I. (2020). The study of generic model set for reducing calibration time in P300-based brain-computer interface. *IEEE Transactions on Neural Systems and Rehabilitation Engineering*.

[27] Yoon J., Lee J., Whang M. (2018). Spatial and time domain feature of ERP speller system extracted via convolutional neural network. *Computational Intelligence and Neuroscience*.

[28] Belkacem A. N., Jamil N., Palmer J. A., Ouhbi S., Chen C. (2020). Brain computer interfaces for improving the quality of life of older adults and elderly patients. *Frontiers in Neuroscience*.

[29] Shao L., Zhang L., Belkacem A. N. (2020). EEG-controlled wall-crawling cleaning robot using SSVEP-based brain-computer interface. *Journal of Healthcare Engineering*.

[30] Chen C., Zhou P., Belkacem A. N. (2020). Quadcopter robot control based on hybrid brain-computer interface system. *Sensors and Materials*.

[31] Chen C., Zhang J., Belkacem A. N. (2019). G-causality brain connectivity differences of finger movements between motor execution and motor imagery. *Journal of Healthcare Engineering*.

[32] Chen C., Li X., Belkacem A. N. (2019). The mixed Kernel function SVM-based point cloud classification. *International Journal of Precision Engineering and Manufacturing*.

[33] Chen C., Chen P., Belkacem A. N. (2020). Neural activities classification of left and right finger gestures during motor execution and motor imagery. *Brain-Computer Interfaces*.

